# The Role of fMRI in the Assessment of Neuroplasticity in MS: A Systematic Review

**DOI:** 10.1155/2018/3419871

**Published:** 2018-12-31

**Authors:** De Giglio Laura, Tommasin Silvia, Petsas Nikolaos, Pantano Patrizia

**Affiliations:** ^1^Department of Human Neurosciences, Sapienza University of Rome, Rome, Italy; ^2^Multiple Sclerosis Centre, Sant'Andrea Hospital, Sapienza University of Rome, Rome, Italy; ^3^IRCCS NEUROMED, Pozzilli, Italy

## Abstract

Neuroplasticity, which is the ability of the brain to adapt to internal and external environmental changes, physiologically occurs during growth and in response to damage. The brain's response to damage is of particular interest in multiple sclerosis, a chronic disease characterized by inflammatory and neurodegenerative damage to the central nervous system. Functional MRI (fMRI) is a tool that allows functional changes related to the disease and to its evolution to be studied in vivo. Several studies have shown that abnormal brain recruitment during the execution of a task starts in the early phases of multiple sclerosis. The increased functional activation during a specific task observed has been interpreted mainly as a mechanism of adaptive plasticity designed to contrast the increase in tissue damage. More recent fMRI studies, which have focused on the activity of brain regions at rest, have yielded nonunivocal results, suggesting that changes in functional brain connections represent mechanisms of either adaptive or maladaptive plasticity. The few longitudinal studies available to date on disease evolution have also yielded discrepant results that are likely to depend on the clinical features considered and the length of the follow-up. Lastly, fMRI has been used in interventional studies to investigate plastic changes induced by pharmacological therapy or rehabilitation, though whether such changes represent a surrogate of neuroplasticity remains unclear. The aim of this paper is to systematically review the existing literature in order to provide an overall description of both the neuroplastic process itself and the evolution in the use of fMRI techniques as a means of assessing neuroplasticity. The quantitative and qualitative approach adopted here ensures an objective analysis of published, peer-reviewed research and yields an overview of up-to-date knowledge.

## 1. Introduction

Neuroplasticity, which is the ever-changing adaptation of the brain to new conditions, is a key factor in the pathophysiology of MS, a central nervous system immunity-mediated disease. MS-related changes, at the level of synaptic transmission, gene expression, and structural and functional organization at different scales, can be promoted or impeded by the action of immunity, inflammation, drugs, and rehabilitation [1]. However, neuroplastic potential appears to be preserved even under widespread damage and can contribute, with the right interventional drive, to restoration and recovery [2]. Functional MRI (fMRI), a noninvasive approach for studying large-scale brain networks that has been used ever since it was introduced to assess MS-related evidence of neuroplasticity, reveals modifications in task condition (t-fMRI), with altered activation or deactivation patterns and the recruitment of additional brain areas being observed in all functional domains [3, 4]. The advent of resting-state condition scanning (r-fMRI) has allowed the assessment of functional connectivity (FC), the identification of specific resting-state networks (RSNs), and the evaluation of relevant functional alterations in MS, both within and between networks [5]. In recent years, both t-fMRI and r-fMRI have been used in cross-sectional and longitudinal statistical designs, usually controlled against healthy subjects, either with or without disease-modifying therapy or rehabilitation interventions, to assess disease-specific changes. Several heterogeneous approaches to data analysis have been developed to apply models from sectors such as time series and source analysis and graph theory, with a priori assumptions or data-driven methods, to search for correlations or causality in the characterization of connectivity. Moreover, an assessment of neuroplasticity at this level of large-scale brain network level needs to be based on correlations with clinical, behavioural, and structural parameters if observations are to be interpreted correctly. Indeed, there is a relevant gap in the interpretation of fMRI changes to demonstrate the adaptive or altered nature of changes. Dysfunctional neuroplasticity may manifest itself at some point in the history of the disease, disrupting global and/or specific brain networks, and may play an important role in overall clinical deterioration [6]. Evaluating whether plastic changes are beneficial or detrimental is highly challenging, owing above all to the complexity and dynamicity of the underlying processes, which may or may not lead to clinical manifestations. This is also the reason why there are as yet no definitive applications for fMRI findings in clinical decision-making, a breakthrough the neurological community has long been awaiting.

The aim of this systematic review is to determine the state-of-the-art importance in fMRI-based assessments of neuroplasticity in relation to MS-induced changes. For this purpose, we considered all the scientific papers published in the literature, analyzing them according to both the technique (t-fMRI or r-fMRI) and study design (cross-sectional, longitudinal, or interventional) adopted.

## 2. Methods

We searched two electronic databases (PubMed and Scopus), according to the Preferred Reporting Items for Systematic Reviews and Meta-Analyses (PRISMA), using the following terms: *plasticity* OR *neuroplasticity* OR *reorganization* OR *reorganisation* OR *compensatory* OR *compensation* AND *multiple sclerosis* AND “*fMRI*.” The last search was undertaken on the 4^th^ of April 2018, and no restrictions were applied to the article type or time period.

To identify further articles, we searched the study references, including references from original articles and reviews that emerged from the search. We are not aware of other studies that fall within the scope of this review except for a paper from our group that has been included in the present analysis.

Once the documents identified in the two databases had been screened to exclude reviews, the two series were checked to exclude double counting. Abstracts were examined to ensure that the eligibility criteria were fulfilled. Any case reports, articles not written in English, or articles that included patients affected by neurological conditions other than multiple sclerosis were excluded. Furthermore, since the aim of this review was to summarize the contribution of fMRI to the study of brain plasticity in MS, we also excluded articles designed to assess fMRI activity in the spinal cord or MS-related plasticity using techniques other than MRI and articles that exclusively evaluated methodological or theoretical aspects. Lastly, we only considered studies performed on adult subjects to exclude articles in which a possible contribution of CNS development to neuroplasticity was considered.

This procedure was carried out by three investigators, two of whom (LDG and ST) independently examined the abstracts; in case of disagreement, a third, more experienced author (PP) made the final decision after examining the whole paper.

Information on selected articles was recorded on an electronic spreadsheet (LDG) and labelled according to the fMRI protocol and study design used. This led to the following six paper categories being identified: t-fMRI cross-sectional, longitudinal, and interventional studies and r-fMRI cross-sectional, longitudinal, and interventional studies.

We applied a customised set of criteria, adapted from those used by Welton and colleagues [7], to assess the scientific quality of the selected papers and to identify any possible sources of bias. A checklist of ten questions was created and a point was assigned for each quality criterion fulfilled; a maximum score of 10 points could thus be attained.

To evaluate the evolution of the methodologies over time, Spearman's correlations were performed between the year of production and paper categories. To compare the quality of the paper categories, we also performed a two-tailed, alpha = 0.05, Wilcoxon test between the entire r-fMRI and t-fMRI groups. Lastly, to define those elements in the quality assessment that improved within the time period considered, Spearman's correlations were performed between the year of production and the average result per year of each question, e.g., in the year 2011, two out of 7 papers received 1 point for question 1, yielding an average of 0.3.

## 3. Results

**Figure 1:**
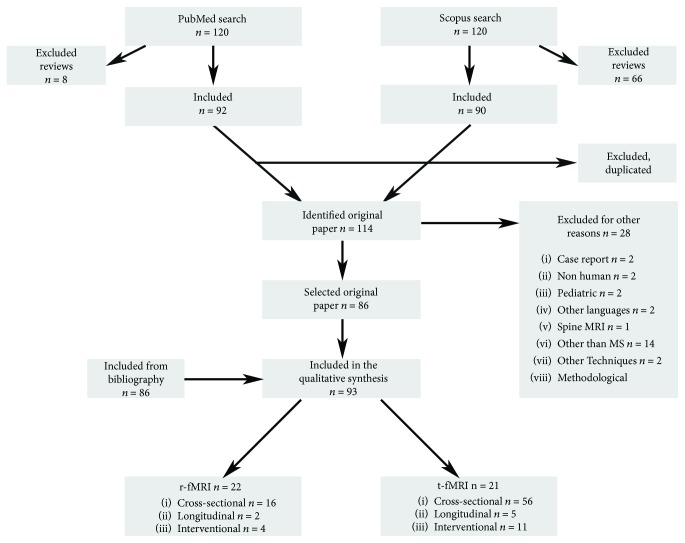
Work flow chart.

The selection process is summarized in Figure 1. As there was never any disagreement in the evaluation of the abstracts, the opinion of the more experienced evaluator was never necessary.

Briefly, we initially found 120 documents in the PubMed database and 156 in that of Scopus. After excluding the reviews, we obtained 92 papers from PubMed and 90 from Scopus. A comparison of the two search results showed that 68 papers were present in both. Therefore, once the duplicates were excluded, a total of 114 abstracts were checked for exclusion criteria and 86 papers were selected.

Lastly, 7 documents were added from the references of selected papers. Ninety-three papers were analyzed in this review and included in the qualitative analysis.

The fMRI images in 71 studies were acquired during the performance of a task: the majority (*n* = 55, 21 of which were based on motor tasks and 34 on cognitive tasks) were cross-sectional studies, 5 were longitudinal, while 11 were based on a pharmacological or training intervention.

The fMRI images in the remaining 22 studies were acquired at rest: 16 were cross-sectional studies, 2 were longitudinal, while 4 were based on a pharmacological or training intervention.

**Figure 2:**
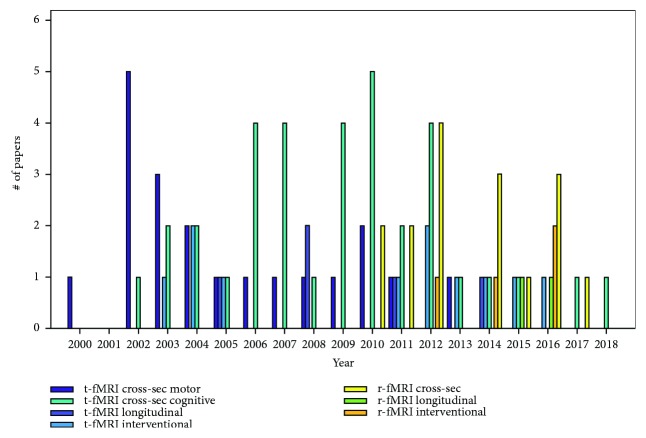
Paper distribution over time. Number of papers published every year from 2000 to 2018, divided according to both the technique (t-fMRI or r-fMRI) and study design (cross-sectional, longitudinal, or interventional).

Cross-sectional fMRI during a motor task was the first type of functional investigation to be performed in 2000 and has been the most commonly used since then, followed in time by cross-sectional cognitive fMRI, longitudinal fMRI, and interventional task-based fMRI. Cross-sectional fMRI during the performance of a cognitive task has produced the largest amount of literature, with a total of 37 papers being published and the peak occurring in 2011. After r-fMRI was introduced in 2011, cross-sectional studies based on this technique peaked in 2012 and longitudinal and interventional investigations took off thereafter (yearly production shown in Figure 2).

**Figure 3:**
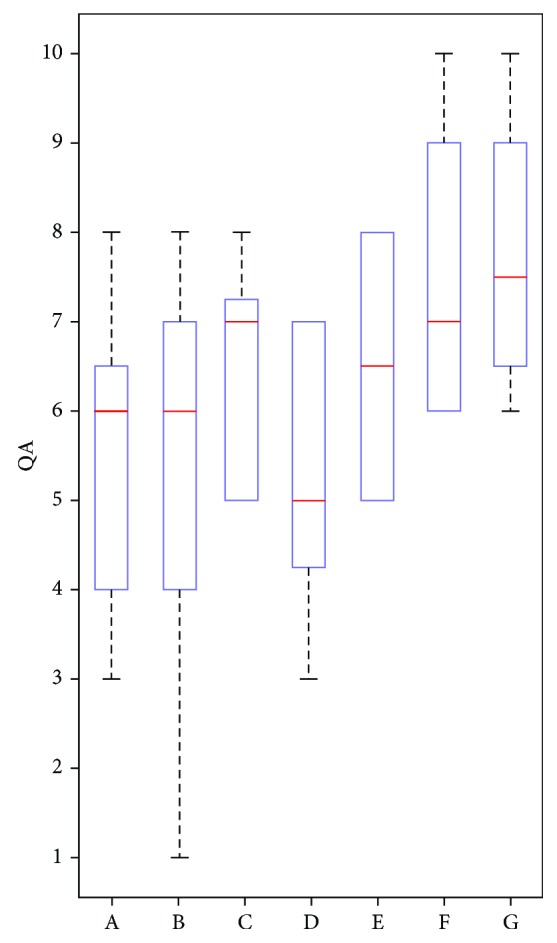
Quality assessment (QA). Distribution of quality scores for each category of papers: blue rectangles represent the 25%-75% quartile range of distribution, red lines the median, black dashed lines the outliers. On X axes: A: cross-sectional motor t-fMRI; B: cross-sectional cognitive t-fMRI; C: longitudinal t-fMRI; D: interventional t-fMRI; E: cross-sectional r-fMRI; F: longitudinal r-fMRI; G: interventional r-fMRI.

Quality assessment showed that cross-sectional t-fMRI studies during the performance of a cognitive task underwent a significant improvement over time (*r* = 0.44, *p* < 0.05 corrected for multiple comparison). Group comparison showed an overall better quality in r-fMRI than in t-fMRI studies (*p* < 10^−4^, Figure 3).

Sample size (Q2: *r* = 0.5, *p* < 0.03), magnetic field strength (Q5: *r* = 0.7, *p* < 0.01 corrected for multiple comparison), use of covariates of no interest in the statistical analysis (Q7: *r* = 0.8, *p* < 10^−4^ corrected for multiple comparison), studies of correlation between functional outcome and clinical scores (Q8: *r* = 0.5, *p* < 0.03), and clear statement of research limits (Q10: *r* = 0.8, *p* < 10^−4^ corrected for multiple comparison) were the checklist criteria that improved over time.

### 3.1. Task-Related fMRI Studies

#### 3.1.1. Cross-Sectional

We identified 55 task-related cross-sectional fMRI studies, comprising 21 studies performed during the execution of a motor task and 34 studies during the execution of a cognitive task (Table 1).

*(1) Motor Task*. A number of different protocols have been used to explore the function of the motor system: scans were generally obtained during a finger flexoextension hand movement; in other studies, scans were obtained during the “thumb-to-finger” movement, consisting in the repetition of the thumb opposition against the index or the sequential opposition of all the fingers against the thumb. In most studies, movements were executed with the dominant hand.

Despite the differences in the task used, the findings in the literature do converge on some key points. Patients exhibited greater cortical activation than healthy subjects (HS), often involving motor areas in both cerebral hemispheres, in all disease forms, i.e., clinically isolated syndromes (CIS) [8–11] and relapsing–remitting (RR) [12, 13], secondary progressive (SP) [14, 15], and primary progressive (PP) [16–18] MS. Studies that evaluated cortical activation in patients who had completely recovered from a single relapse provided new insights into functional reorganization after acute brain damage by showing a wider motor activation that also involves the ipsilateral hemisphere compared with HS [8, 19].

The comparison of different MS phenotypes revealed various patterns of motor activation, with a prevalent functional lateralization in CIS, a bilateral pattern in RR MS, and more extended activation, even involving areas outside the motor system, in SP MS [20]. In the early forms of MS, patients generally performed the motor task as well as HS; this ability tended to disappear as the disease progressed. However, data obtained by using passive movements, which are not affected by patient performance and disability, confirmed that MS patients exhibit greater motor activation than HS, with more extensive activation being observed in SP MS than in RR MS [21]. Several studies addressed the issue of the relationship between functional and structural changes. Structural damage was quantified by means of various MR techniques, i.e., conventional T2- and T1-weighted images to assess lesion burden and spectroscopy or diffusion tensor imaging (DTI) to assess apparently normal brain tissue integrity. Generally, increased sensorimotor activation was found to correlate with the severity of tissue damage, especially if calculated in specific tracts of white matter, i.e., the corticospinal tract [2, 8], with more pronounced axonal damage as calculated by spectroscopy [13, 22] or with widespread microstructural tissue damage as measured by DTI [14, 15].

*(2) Cognitive Task*. There were even greater variations between the paradigms adopted in studies designed to explore cognitive functions owing to the different cognitive domains explored. For example, the modified versions of the Paced Auditory Serial Addition Test (PASAT, [23–30]) or of the N-back test [31–36] were commonly used for the assessment of working memory; decision-making abilities were tested by using an adapted version of the Iowa gambling task [37]; cortical activation related to memory was studied by means of a verbal memory and episodic memory encoding paradigm [38, 39]. Lastly, some specific paradigms were used to test activation during tasks that became increasingly complex and/or induced cognitive fatigue, such as adaptation of the Go/No Go test [40, 41].

The majority of the studies reported a greater extension of cortical activation during the performance of cognitive tasks in patients with no or minimal cognitive impairment compared with HS [23, 26, 27, 42–44]. Increased cortical activation during attention and memory tasks was mainly observed in those patients who performed as well as HS, it being less evident in poor performers [26]. Differences were consistently reported between patients with and without cognitive impairment, with the former exhibiting a lower degree of activation in the hippocampal network during a memory task [38]. Similarly, Penner et al. showed that attention tasks of varying complexity induced additional activation in MS patients compared with HS; however, severe MS patients did not display any additional activation in the prefrontal structures or in the premotor cortex, thereby suggesting that the compensatory mechanisms had become exhausted [45].

Furthermore, patients with RR MS displayed greater cortical activation than CIS patients or HS as the task difficulty increased. By contrast, patients with SP MS displayed only a slight increase in brain activation as task difficulty increased, thus pointing to the presence of a limited functional reserve [41]. Similarly, Rocca et al. showed that, with increasing task difficulty, cognitively impaired MS patients exhibited a lower activation in several cortical areas when compared with HS and cognitively preserved MS patients [36].

Several studies also reported an association between abnormal fMRI activation and measures of brain structural damage, which was evaluated as lesion load [26, 32], as loss of integrity of matter measured using DTI, or as brain atrophy [34, 41].

#### 3.1.2. Longitudinal Studies

Few studies have used t-fMRI to investigate the evolution of brain plastic changes over time (Table 2). While patients with MS exhibited greater bilateral activation than HS at the baseline fMRI during a motor task, they exhibited a reduction in functional activity in the ipsilateral sensorimotor cortex and in the contralateral cerebellum after a 20-month follow-up period. Decreased activation in the ipsilateral motor cortex inversely correlated with age, progression of T1 lesions, and occurrence of new relapses during the follow-up period [46]. This result was interpreted as a return of functional activation towards a more normal pattern in those patients with a more benign clinical course.

Similarly, Mezzapesa et al. demonstrated that early cortical changes after an acute motor relapse due to a pseudotumoral lesion consisted in the recruitment of pathways in the ipsilateral hemisphere; functional recovery in the contralateral motor areas and decreased ipsilateral activation were associated with a good recovery [47].

By contrast, Audoin et al., who studied the association between activation changes over time and cognitive performance, found a significant correlation between extended activation in the prefrontal cortex after a 12-month follow-up period and improvement in cognitive functions. Therefore, unlike findings obtained for the motor system, an improvement in cognitive performance over time was associated with increased activation of the related cortical areas [48].

#### 3.1.3. Interventional Studies

Eleven studies have investigated intervention-induced changes in cortical activation (Table 3).

The fact that changes in cortical activation may be induced by the administration of drugs suggests that neuroplasticity may be pharmacologically modulated. Parry showed that the acute administration of rivastigmine changed the activation pattern in MS patients in such a way as to make it more similar to that observed in HS [49]. Mainero et al. reported that the administration of a single dose of 3,4-diaminopyridine increased activation in the sensorimotor cortex and supplementary motor area during the performance of a motor task [50]. Lastly, Tomassini et al. investigated whether plasticity could be enhanced by means of a pharmacological intervention to reduce inflammation. They demonstrated that short-term plasticity is enhanced by the administration of interferon beta for a period of 12 weeks [51].

Short-term motor training reduced activation in the contralateral sensorimotor cortex in HS though not in MS patients [52]. By contrast, a larger study by Tomassini et al. showed that brain plasticity persists after visuomotor training in MS patients. They demonstrated that short-term training reduced activation in a larger number of cortical areas in MS patients than in HS. After a two-week practice period, there were significant decreases in visuomotor task-related activation in the occipital cortex in HS and in both the occipital and parietal cortices in MS patients [2]. This result is in keeping with changes in cerebral activation following a cognitive intervention or rehabilitation training reported in other studies. Indeed, increased activation in the cerebellum and in the superior parietal lobule has been reported after cognitive training; furthermore, the fact that the changes in brain function positively correlated with an improvement in clinical outcomes points to a cause-effect role of such interventions [53]. The application of an individualized rehabilitative protocol consisting in neurophysiological, sensorimotor learning and adaptation therapy led to a reduction in cerebellar activation in the supplementary motor cortex and an enlargement in the primary sensorimotor cortex [54]. Although studies on smaller numbers of patients also demonstrated changes in cortical activation, the limited size of the samples prevented the identification of both the specific regions involved and possible correlations with clinical outcomes [52, 55, 56].

### 3.2. Resting-State Studies

#### 3.2.1. Cross-Sectional Studies

Sixteen studies have been performed using fMRI acquisition at rest and a cross-sectional design (Table 4). Since ranges of different data analysis methods were applied, the results of the various studies cannot always be compared. We identified three studies that used amplitude fluctuation (ALFF), six studies that used independent component analysis (ICA), three studies that used seed-based methods, and three studies that used the graph theory approach.

An r-fMRI study performed after the first demyelination episode revealed lower ALFF in MS patients than in HS [57]. Other studies instead found that ALFF in MS patients increases in the thalami and several cortical regions and that these changes correlated with disability [58, 59].

Using ICA, Roosendaal et al. showed a higher degree of synchronization in patients with CIS than in either HS or MS patients in six RSNs, including the default mode network (DMN) and sensorimotor network, whereas no significant functional differences were detected between MS patients and HS. Furthermore, FC in several RSNs in the group of MS patients was found to decrease gradually. This decrease correlated with increasing damage, thereby suggesting that cortical reorganization in the RSNs is a phenomenon that occurs early in the disease course of MS and that tends to become exhausted as the disease progresses and damage accumulates [60]. Faivre et al. reported that FC in seven RSNs was significantly increased in MS patients compared with HS. A negative correlation was also reported between the multiple sclerosis functional composite, a measure of global disability, and increased FC within the dorsal frontoparietal, right frontoparietal, and the prefronto-insular networks [61]. A gender difference has also been reported to parallel differences in cognitive performance: decreases in FC and network efficiency in male patients were found to correlate with reduced visuospatial memory [62].

Alterations in the DMN have also been reported to correlate with fatigue and clinical measures of disability [63, 64]. Reduced activity in the anterior component of the DMN has been demonstrated in the progressive forms of MS compared with HS, with a more pronounced DMN alteration in patients with cognitive impairment being correlated with DTI alterations in the corpus callosum and cingulum. MS patients with fatigue displayed increased FC in the posterior component of the DMN and in the primary motor cortex and reduced FC in the anterior component of the DMN and in the supplementary motor cortex of the SMN compared with patients without fatigue [64].

The visual network has also been explored. MS patients exhibited reduced FC in the peristriate visual cortex, bilaterally, compared with HS. Patients with a history of visual symptoms displayed greater FC in the extrastriate cortex and right lateral middle occipital gyrus as well as reduced FC in the right inferior peristriate cortex. These differences correlated with the number of episodes affecting the visual system though not with regional grey matter atrophy [65]. Liu et al. studied the FC in CIS patients without conventional brain lesions and in MS patients. Compared with HS, CIS patients displayed reduced connectivity in the visual areas and increased connectivity in other areas, above all those located in the temporal lobes; patients with MS exhibited more widespread increased FC, especially in the deep grey matter, than either CIS patients or HS. The baseline FC levels were found to be higher in patients who had a clinical relapse over the subsequent 5 years (and who thus converted to MS patients) than in those who remained stable over the same period of time [66].

Two studies used a seed-based method approach to study thalamic FC. The first of these studies revealed the coexistence of areas of increased FC and decreased FC in MS patients when compared with HS. Interestingly, a negative correlation was observed between thalamic FC and cognitive performance, which indicates that the greater the thalamic connectivity is with some cerebral areas, the more severe the cognitive impairment is [67]. Liu et al. reported decreased FC between the thalamus and several brain regions, including the right middle frontal and parahippocampal gyri and the left inferior parietal lobule. The authors of that study also reported greater intra- and interthalamic FC in the MS group, which negatively correlated with disease duration, than in HS [68]. Another r-fMRI study based on a seed analysis evaluated the relationship between FC in the sensorimotor network and upper limb motor disability. MS participants in whom motor skills were preserved displayed greater FC in structurally intact visual information processing regions than MS patients in whom motor skills were impaired. By contrast, motor-impaired MS participants displayed weaker FC in the sensorimotor and somatosensory association cortices and more severe structural damage throughout the brain [69].

Brain network FC was studied in a large cohort of patients with MS by applying graph theoretical analysis to r-fMRI. Global network properties were abnormal in MS patients and helped to distinguish cognitively impaired MS patients from HS [70]. The eigenvector centrality mapping (ECM) method, a graph analysis technique that ranks the importance of brain regions according to their connectivity patterns, was applied to a large sample of MS patients. ECM values in MS patients were found to be increased in the bilateral thalamus and posterior cingulate areas and decreased in the sensorimotor and ventral stream areas, with sensorimotor ECM decreases being related to higher disability [71].

### 3.2.2. Longitudinal Studies

Few studies have assessed changes in FC over time (Table 5). Droby et al. assessed the effects of acute and chronic lesions on FC in RR MS patients by studying nine RR MS patients over five time points at 8-week intervals; one of the nine patients developed an acute WM lesion. They concluded, despite the small sample size, that lesion-related network changes may occur as a result of reorganization processes following the initial appearance of an acute lesion [72].

A subsequent study by Faivre et al. applied graph theoretical analysis of r-fMRI to 38 patients with RR MS and 24 HS. The authors found higher baseline levels of long-range and short-range brain FC in patients than in HS. At the 2-year follow-up, patients exhibited a reduction in FC that correlated with disability progression, thus suggesting that reduced connectivity reflects the exhaustion of compensative proprieties [73].

### 3.2.3. Interventional Studies

We identified four r-fMRI studies that investigated changes in FC after training, rehabilitation, or drug administration (Table 6). Short-term changes in FC associated with a simple repetitive motor task were investigated in early RR MS patients. After 25 minutes of a repetitive thumb flexion task, significantly greater cerebellar FC was observed in the cerebellar network of MS patients though not in that of HS. By contrast, FC in the sensorimotor network increased in both groups after the task, with no significant between-group differences. Sensorimotor and cerebellar FC were intercorrelated after the training in patients only [74].

Two interventional studies evaluated FC changes associated with cognitive rehabilitation. When Filippi et al. applied the ICA method in the first of these studies, they found that, after 12 weeks of rehabilitation, MS patients exhibited changes in FC in several cortical areas and a significant association between FC changes and cognitive improvement [75].

De Giglio et al. used a seed method, considering the thalamus as a region of interest, to evaluate the thalamic FC changes induced by an 8-week video game-based training program. After rehabilitation, FC in MS patients increased in the cingulum, in the precuneus and bilaterally in the parietal cortex, and decreased in the cerebellum and in the left prefrontal cortex. Interestingly, thalamic FC changes in these regions significantly correlated with cognitive improvement [76].

Another interventional randomised study assessed the effect of intermittent theta-burst stimulation delivered over the primary motor cortex on MS-related spasticity and on the topology of brain functional networks using a graph theoretical approach [77]. The improvement in spasticity was greater in patients treated with intermittent theta-burst stimulation than in the control group. Indeed, the intermittent theta-burst stimulation on the motor cortex induced an improvement in plasticity paralleling the increase of connectivity between motor cortices of the two hemispheres.

## 4. Discussion

This review documents the extensive use of fMRI as a means of assessing neuroplasticity in MS over the last 18 years and provides new insights into the pathophysiology of this disease. It also shows that the quality of data acquisitions, data analyses, and study designs has steadily increased since pioneering studies in the early 2000s.

### 4.1. Interpretation of fMRI Data: Adaptive or Maladaptive Plasticity in MS

t-fMRI studies, performed with either motor or cognitive tasks, have generally demonstrated greater cortical activation in MS patients than in HS, which has been interpreted as an attempt to compensate for damage [19, 24–26, 78]. More recent studies have demonstrated that the activation pattern varies according to the MS phenotype, with more extensive activation being observed in the progressive form of the disease [14–18, 79, 80]. Furthermore, the increase in activation has been found to correlate with measures of tissue damage [25, 81].

Taken together, these data suggest that the increased activity observed during a task may not only represent compensation for specific damage but also be a marker of disease severity. The main concept that emerges from t-fMRI studies is that adaptive plasticity is a finite process that occurs in the early phases of MS and is aimed at maintaining normal function despite the structural damage but becomes exhausted as the disease progresses. This line of reasoning is supported by the first r-fMRI study [60], which described increased FC in several RSNs in CIS patients though not in MS patients and thus confirmed that cortical functional reorganization is an early and finite phenomenon in MS.

Data based on r-fMRI confirm that functional reorganization is a dynamic process in MS, with changes in FC being observed in different disease stages [57–59]. r-fMRI studies using ICA have highlighted an association between disability measures and reorganization of various RSNs [61, 63–65]. However, the results yielded by r-fMRI have not been univocal: some studies detected an association between increased FC in some RSNs and clinical preservation, whereas others detected an association between increased FC and poor performance [61, 67, 82]. Increased FC may thus represent both adaptive and maladaptive plasticity, and correlations with clinical measures appear to be essential to be able to interpret data correctly. Since it has even been suggested that adaptive and maladaptive mechanisms may coexist [67], it may be necessary to investigate the brain as a whole in large-scale fMRI studies and not to limit the study of functional changes to single RSNs: this might lead to the identification of cooccurring patterns of FC changes as well as of those associated with a potential beneficial clinical effect.

Some specific aspects within this framework deserve greater attention. The first is that the DMN is the RSN that has been investigated most extensively: DMN dysfunction has been reported and associated with cognitive impairment [63, 64]. In particular, alterations in the anterior component of the DMN are considered to be responsible for the accumulation of cognitive deficits in patients with progressive MS [83]. Moreover, functional changes in the DMN rather than in the sensorimotor network have been shown to be related with fatigue in MS [64].

Thalamic FC has also been found to correlate with cognitive performance in MS. Tona et al. described altered thalamic FC in RR MS, showing that there is a significant association between decreased cognitive performance and areas of increased thalamocortical FC and thus suggesting that neuroplasticity changes are unable to compensate for tissue damage and to prevent cognitive dysfunction [67]. Subsequently, Liu et al. reported lower thalamic FC with several cortical regions and greater interthalamic FC in patients with RR MS than in HS. In their study, interthalamic FC significantly correlated with disease duration, thereby pointing to an adaptive role of thalamic FC, which gradually declines as the disease progresses [68].

### 4.2. Application of fMRI to the Study of Postdamage Recovery and of Functional Reserve

Longitudinal studies have shown that fMRI can be used to investigate both functional reorganization after acute damage and the exhaustion of plasticity mechanisms over time.

Two longitudinal studies designed to explore the motor system yielded similar conclusions. In a t-fMRI study by Pantano et al., the decrease in ipsilateral activation that enhances motor function lateralization was limited by advanced age, T1 lesion accumulation, and new relapses [46], whereas in a study by Mezzapesa et al., a return to more lateralized motor activation was observed in a subset of patients who recovered from a motor relapse [47]. Both these studies suggest that brain damage is associated with greater cortical activation and that subsequent clinical recovery is accompanied by the return of brain activity to a pattern more similar to that observed in normal conditions.

Another concept supported by longitudinal fMRI studies is that plasticity mechanisms may become exhausted over time. When Faivre et al. applied graph theoretical analysis to the study of FC over two years, they found that reduced connectivity in patients correlated with disability progression [73].

fMRI has also been used to investigate functional adaptation by studying changes in cortical activation and FC after short training protocols. These studies revealed significant differences between MS patients and HS [2, 74], showing that brain plasticity is still present in MS patients but is characterized by peculiar activation dynamics and FC changes. However, the consistency of these short-term functional changes warrants investigation over longer periods of time to confirm their translational potential for clinical application.

### 4.3. Application of fMRI to Interventional Studies

FMRI was first applied to interventional studies to investigate short-term functional changes induced by drugs [49, 50]. Only more recently have the long-term pharmacological effects on neuroplasticity been investigated: Tomassini et al. applied t-fMRI to study the extent to which brain activity is modified by the administration of immunomodulating/anti-inflammatory therapy for approximately 12 weeks [51].

Rehabilitation is another type of plasticity-inducing intervention that may be explored by means of fMRI. It is possible, despite the small number of studies conducted to date, to draw some conclusions in this regard. First, the results of t-fMRI studies suggest that the changes vary according to the rehabilitative program adopted and may involve more extensive areas if the interventional protocol is designed to target several neurological functions [52, 53]. These results have been confirmed by r-fMRI studies on cognitive rehabilitation and on intermittent theta-burst stimulation of the primary motor cortex. Changes in RSN connectivity and reorganization of the primary motor cortex may represent a functional substrate for cognitive improvement and spasticity, respectively [76, 77].

Another noteworthy finding that has emerged from rehabilitation studies is the frequent involvement of cerebellar areas induced by both cognitive and personalised rehabilitative programs [53, 75, 76]. These data suggest that rehabilitative intervention may be able to play a crucial role in this region, which is known to be involved in learning processes [84].

It should be borne in mind that the correlation between the fMRI changes observed and clinical outcomes is needed not only to be able to interpret the data correctly but also to provide proof that any changes are related to the effectiveness of the intervention.

### 4.4. Improving Methodologies

The interest in neuroplasticity in MS that emerges from the literature has clearly evolved between 2000 and 2018. Indeed, the initial focus on cross-sectional fMRI during a motor task gradually decreases in favor of cognitive task-based studies, which are subsequently followed by cross-sectional resting-state investigations that reflect the growing interest in spontaneous brain activity. The focus on r-fMRI is very likely due to an inherent feature of such studies, i.e., that they are free of the confounding effect of task performance and patient disability and can therefore be performed more easily. Longitudinal and interventional fMRI studies have instead yielded a more limited number of publications than cross-sectional studies, with one paper being published approximately every 1 to 2 years, probably owing to the difficulties involved in sample enrollment and study design.

We found an improvement in the quality of the studies over time, though this improvement is limited to cross-sectional t-fMRI during a cognitive task and to cross-sectional r-fMRI, probably as a result of the high number of publications in these two paper categories (Table 7).

The fact that the quality of r-fMRI studies is higher than that of t-fMRI studies may be due to the improvement and refining of both the technology [85] and methods of analysis in recent years.

## 5. Conclusion

fMRI has, over the years, provided important insights into plasticity and functional reorganization related to MS damage. Recent studies suggest that fMRI can detect functional changes related to clinical improvement and that some brain regions may play a crucial role in recovery. This may in the future help to optimize the use of fMRI in interventional trials, though more longitudinal studies based on larger patient samples are needed to achieve this result. Although the possibility of using fMRI as a tool for clinical decision-making in individual patients seems a long way off, some new analysis techniques to measure plasticity reserves appear to be promising applications of fMRI measures as predictors of clinical outcome.

## Figures and Tables

**Table 1:** Cross-sectional t-fMRI papers.

Author(s) (year)	Sample size	Age (years)	MS duration (years)	EDSS	Task(s) (setting and schedule)	fMRI main result(s)^∗^	Clinical correlation(s)	Structural correlation(s)
Sensorimotor task studies								
Reddy et al. (2000) [22]	9 MS 8 HS	**—** **—**	*11.6* (3.3–23.2)	3.0 (0.0–6.5)	4-finger flexion extension	Greater activation of the ipsilateral SMC	—	Negative correlation with N-acetyl-aspartate concentration
Filippi et al. (2002) [16]	26 PP 15 HS	*50.1* (34.0–68.0) *48.3* (34.0–62.0)	10.0 (2.0–28.0)	5.5 (2.0–8.0)	4-finger flexion extension	Greater activation in the ipsilateral cerebellum, bilaterally in the STG, ipsilaterally in the MFG, contralaterally in the insula/claustrum	—	Positive correlation with the severity of brain and spine structural damage
Pantano et al. (2002) [19]	10 CIS 10 HS	*32.0* (21.0–51.0) *31.0* (8.0)	*1.8* (0.5–4.0)	1.25 (0.0–2.5)	Finger-to-thumb opposition	Greater number of activated foci in the bilateral hemispheres	Positive correlation with disease duration	Positive correlation with T1-LL in the corticospinal tract
Reddy et al. (2002) [13]	14 RR 8 HS	—	—	2.0 (0.0–7.5)	4-finger flexion extension, active 1-finger tapping, active and passive	Greater activation distinctively produced by disability or tissue damage	Positive correlation with disability	Negative correlation with N-acetyl-aspartate concentration
Pantano et al. (2002) [8]	20 CIS 10 HS	*31.7* (8.0) *31.0* (8.0)	*24.3* (14.0) *23.9* (20.0)	*1.25* (0.8) *0.45* (0.6)	Finger-to-thumb opposition	Greater activation in CIS patients who had recovered from a motor deficit than in those who recovered from an optic neuritis and HS	No significant correlation with EDSS	Positive correlation with T1- and T2-LL
Rocca et al. (2002) [17]	30 PP 15 HS	*50.4* (34.0–68.0)	10.0 (2.0–28.0)	5.5 (2.0–8.0)	4-finger flexion extension Foot flexion extension	Greater activation	—	Positive correlation with T2-LL
Rocca et al. (2003) [14]	13 SP 15 HS	*48.5* (30.0–59.0) *48.3* (34.0–52.0)	*13.0* (5.0–35.0)	4.5 (1.5–7.5)	4-finger flexion extension Foot flexion extension	Greater activation for both tasks	—	Positive correlation with MD and FA of NA-WM and NA-GM
Rocca et al. (2003) [9]	16 CIS 15 HS	*31.7* (22.0–43.0) *33.6* (21.0–45.0)	<3 months (mean 34 days)	0.0 (0.0-1.0)	4-finger flexion extension	Greater activation	—	Positive correlation with the concentration of N-acetyl-aspartate in the whole brain
Rocca et al. (2003) [80]	12 RR 12 HS	38.0 (22.0–53.0) 37.3 (26.0–59.0)	2.5 (2.0–17.0)	1.5 (0.0–6.0)	4-finger flexion extension	Greater activation in the bilateral cortex and contralateral thalamus; *lower* in the contralateral parietooccipital GM and ipsilateral SMC		Negative correlation with MD magnitude and positive correlation with MD location
Filippi et al. (2004) [12]	16 RR 16 HS	*36.4* (18.0–60.0) *34.6* (24.0–62.0)	7.0 (2.0–17.0)	1.0 (0.0–3.0)	4-finger flexion extension Object manipulation	Greater activation in the SMA, SII, R cerebellum, SPG, and IFG Additional areas of activation during object manipulation	—	—
Filippi et al. (2004) [86]	16 CIS 15 HS	*31.7* (22–43) *33.6* (21–45)	34 days (18.0–64.0)	0.0 (0.0-1.0)	4-finger flexion extension Foot flexion extension	Greater activation of the contralateral SMC, SII, and IFG	—	No significant results
Rocca et al. (2005) [20]	16 CIS 14 RR ND 15 RR MD 12 SP	*31.7* (22–43) *37.6* (24.0–54.0) *35.4* (18.0–52.0) *50.0* (30.0–59.0)	*0.1* (0.1–0.2) *9.5* (2.0–22.0) *8.0* (2.0–17.0) *17.0* (5.0–35.0)	0.0 (0.0-1.0) 0.0 (0.0-1.0) 1.0 (1.0–3.0) 4.5 (1.5–7.5)	Four-finger flexion extension	Cortical activation varies with disease phenotype	No significant results	—
Ciccarelli et al. (2006) [18]	PP 13 HS 16	*46.6* (11.3) *37.3* (11.9)	*8.69* (7.49)	4.0 (3.0–6.5)	Foot flexion extension, active and passive	Greater activation in the STG, Rolandic operculum, and putamen during passive movement	Negative correlation with EDSS (active movement)	Negative correlation with T2-LL (passive movement)
Wang and Hier (2007) [87]	15 MS 10 HS	*41.9* *45.8*	11.8	3.7 (1.0–8.0)	4-finger flexion extension	Greater activation in R PMC and R cognitive areas	—	Positive correlation with T2-LL
Wegner et al. (2008) [88]	56 MS 55 HS	*35.0* (20.0–53.0) *30.0* (19.0–48.0)	*6.7* (1.0–21.0)	2.0 (0.0–7.5)	Hand tapping	Greater activation	Positive correlation with age and manual dexterity	
Rocca et al. (2009) [79]	MS 61 HS 74	*35.7* (7.4) *30.7* (7.1)	*7.8* (5.3)	2.5 (0.0–7.5)	4-finger flexion extension, DH	Different effective connectivity	No significant correlation with EDSS	Negative correlation with T2-LL
Harirchian et al. (2010) [10]	CIS 26 HS 28	*29.0* (6.48)	—	—	4-finger flexion extension Foot flexion extension	Greater activation	—	—
Rocca et al. (2010) [15]	17 BMS 15 SP 17 HS	*48.5* (38.0–63.0) *48.6* (35.0–65.0) *50.3* (36.0–68.0)	24.0 (15.0–35.0) 22.0 (15.0–32.0)	2.0 (1.0–3.0) 6.5 (5.5–8.0)	4-finger flexion extension	Grater activation in BMS only in the contralateral SMC Additional areas of activation in SP	All MS: negative correlation with EDSS in the R cerebellum	Correlation in all MS with T2-LL, MD, and FA in NA-WM.
Rico et al. (2011) [11]	8 CIS 10 HS	*30.0* (23.0–5.0) *29.0* (22.0–9.0)	*0.3* (0.1–0.7)	1.3 (0.0–3.0)	4-finger flexion extension	Greater activation in the ACC	—	Positive correlation with T2-LL
Petsas et al. (2013) [21]	13 RR 18 SP 15 HS	*37.8* (10.4) *49.8* (6.4) *41.7* (9.0)	*7.6* (5.8) *21.9* (8.6)	1.5 (1.0–3.0) 6.0 (6.0–6.5)	Passive four-finger flexion extension	Progressive extension of ipsilateral motor activation and different deactivation of posterior cortical areas according to phenotype	—	Correlation with T2 and T1 lesion volume
Faivre et al. (2015) [89]	13 early MS 14 HS	*32.0* (21.0–43.0) *30.0* (20.0–51.0)	— —	1.0 (0.0–3.0) —	4-finger flexion extension Resting-state fMRI	Greater activation in the R PFC Higher mean FC of the nondominant motor network		—
Cognitive Task Studies								
Staffen et al. (2002) [23]	21 RR 21 HS	*33.5* (7.5) *31.8* (7.4)	—	—	PVSAT	Greater activation in the frontal, parietal, and cingulate cortexes	—	—
Audoin (2003) et al. [24]	10 CIS 10 HS	*31.6* (7.57) *26.1* (7.88)	*0.57* (0.28)	*1.25* (0.0–2.00)	PASAT	Greater activation in the R frontopolar cortex, bilateral lateral PFC, and R cerebellum	No significant results	No significant results
Penner (2003) et al. [45]	14 MS 7 HS	*45.8* (31.0–59.0) matched	11.4 (3.0–24.0)	3.3 (1.0–6.0)	Attention	Greater and more extended activation, not significant in more severe patients	—	—
Mainero et al. (2004) [26]	22 RR 22 HS	*30.5* (22.0–50.0) matched	9.0 (1.0–16.0)	1.5 (1.0–3.5)	PASAT; memory recall task	Greater and more extended activation, more significant in good than in poor performers	No significant results	Positive correlation with T2-LL
Saini et al. (2004) [90]	14 RR 11 HS	37.0 (18.0–52.0) 37.0 (27.0–43.0)	3.6 (8.0)	1.0 (1.0–2.5)	Writing	Greater activation in the R PMC	No significant results	No significant results
Audoin et al. (2005) [25]	18 CIS HS 18	*29.5* (7.0) *25.3* (6.3)	6.6 (4.94) months	1.0 (0.0–2.0)	PASAT	Greater activation in the lateral PFC (bilaterally in good performers, only R in poor performers)	—	Negative correlation with tissue damage in R PFC
Cader et al. (2006) [31]	21 RR 16 HS	39.0 (22.0–55.0) 39.0 (23.0–51.0)	6.0 (1.0–20.0)	2.0 (0.0–6.0)	N-Back	Lower activation in the SFG and ACC; smaller activation increases with greater task complexity	No significant results	No significant results
Forn et al. (2006) [27]	15 RR 10 HS	*32.7* (8.5)	—	*2.13* (0.0–4.0)	PASAT	Greater activation in the L PFC	—	—
Rachbauer et al. (2006) [28]	9 CIS 9 RR 18 HS	*29.5* (5.8) *28.2* (5.3) *26.4* (5.4)	*17.5* (24.2) months	0.0 (0.0-1.0) 0.0 (0.0–2.0)	PVSAT	Greater activation in the hippocampal and parahippocampal areas CIS vs RR and HS: greater activation in the ACC	—	—
Sweet et al. (2006) [32]	15 RR 15 HS	*47.3* (6.8) *48.1* (6.3)	*21.4* (4.6)	1.5	N-Back (*n* = 1, 2, 3)	1-Back: greater activation in the PMC, SMA, and DLPFC; 2-,3-Back: lower activation in the L SFG, cingulate, and parahipp. gyri	Positive correlation of difficulty level in the anterior cortex	Positive correlation of 1-back activity with T2-LV
Forn et al. (2007) [33]	17 RR 10 HS	— Matched	—	*1.65* (0.0–4.0)	N-Back	Greater activation bilaterally in the IFG and insula	—	—
Morgen et al. (2007) [42]	19 RR 19 HS	*32.4* (8.2) *31.7* (7.5)	*20.0* (17.1)	1.5 (1.1)	Delayed recognition (encoding, maintenance, and recognition)	Encoding: no significant differences Maintenance, recognition: greater activation in L IPL	Correlation with PASAT	Positive correlation with GM atrophy
Nebel et al. (2007) [91]	6 RR + *D* 6 RR-*D* 6 HS	*34.3* (6.5) *28.8* (6.9) *33*.0 (5.0)	8.5 (4.0–11.0) 6.0 (3.0–6.0)	3.0 (2.0–5.0) 2.5 (1.5–2.5)	Attention (focused, divided)	(*D* = attention deficit) RR + *D* vs HS: lower activation RR-*D* vs HS: not significant	—	—
Prakash et al. (2007) [29]	24 RR	*44.7* (29.0–53.0)	*8.0* (1.0–18.0)	*2.6* (1.8)	PVSAT	Activation of prefrontal, parietal, temporal, and occipital regions in response to the PVSAT	Peak oxygen consumption correlated positively in the R IFG-MFG and negatively in the ACC	—
Prakash et al. (2008) [43]	24 RR 15 HS	*45.86* *44.74*	*8.0* (5.1)	2.6 (1.7)	Eriksen flanker task (congruent, incongruent, and baseline)	Incongruent > baseline: greater activation in the R PFC Incongruent > congruent: greater activation in the bilateral IFG	Reaction time positively correlated with incongruent condition activation in the R IFG	—
Bonzano et al. (2009) [30]	23 RR 18 HS	*32.5* (4.2)	*6.9* (3.2)	*1.6* (0.0–3.0)	PVSAT vs visual (control) task	No group comparison reported	—	—
Passamonti et al. (2009) [44]	12 RR 12 HS	*29.3* (8.1) *28.7* (5.1)	*4.3* (2.8)	1.5 (1.0–2.5)	Emotion evoking (photos of faces) vs neutral (shapes)	Greater activation in the ventrolateral PFC Lower FC between the L amygdala and PFC	—	—
Pierno et al. (2009) [92]	15 RR 15 HS	*30.6* (19.0–44.0) *34*.0 (24.0–54.0)	*16.2* (9.2)	*1.5* (1.0–3.0)	Hand-grasping observation	Greater activation	—	—
Rocca et al. (2009) [81]	15 BMS 19 HS	*44.0* (35.0–61.0) *41.7* (34.0–60.0)	20 (20–30)	2.0 (1.0–3.0)	STROOP	Greater EC between the SMC and R IFG and R cerebellum; lower with the ACC	Positive correlation with disease duration	Correlations of average FA/MD with EC
Smith et al. (2009) [93]	10 MS 10 HS	*44.0* (8.72) *45.1* (9.42)	<3.0	—	Go/No Go	Greater activation	—	—
Bonnet et al. (2010) [40]	15 RR 20 HS	*35.4* (10.26) *32.5* (9.77)	*29.8* (13.5)	2.5 (0.0–6.0)	Go/No Go (complex, initial), tonic alertness	More extent activation; lower and less extent for more complex tasks	Correlation with response times	Positive correlation with lower mean NA-BT in the MTR
Helekar et al. (2010) [94]	16 RR 18 HS	*39.6* (2.6) *36*.0 (2.2)	7.0 (2.0–15.0)	2.0 (1.0–6.0)	STROOP; Wisconsin Card Sorting task	No significant results	Positive correlation for age with network sizes and spatial extent None with EDSS or disease duration	
Rocca et al. (2010) [34]	16 PP 17 HS	*49.7* (39.0–68.0) *49.9* (26.0–63.0)	10.0 (4.0–21.9)	6.0 (3.0–7.0) —	N-Back	Greater activation with differences between CI and CP CI vs CP: greater activation in the L PFC and IPL; lower in the bilateral SII, cerebellum, and R insula	Positive correlation with composite cognitive score	Negative correlation with T2-LL in the PFC; positive in the SII
Amann et al. (2011) [35]	15 MS 15 HS	*37.6* (6.8) *33.9* (7.6)	*5.9* (3.6) —	*2.3* (1.3) —	Alertness task N-Back (*n* = 1, 2, 3)	Greater activation in simple tasks and greater deactivation at the highest task load	—	—
Jehna et al. (2011) [95]	15 RR 15 HS	*29.5* (9.6) *30.3* (10.6)	*7.3* (6.5) —	2.0 (0.0–3.5) —	Facial recognition of emotion	Greater activation in the PCC and precuneus for anger or disgust; in the occipital fusiform gyri, ACC, and IFG for neutral	—	No significant results
Loitfelder et al. (2011) [41]	10 CIS 10 RR 10 SP 20 HS	*33.4* (10.5) *32.5* (7.5) *46.5* (8.8) *34.0* (8.1)	*1.1* (1.0) *4.7* (4.1) *16.2* (7.0) —	*0.5* (0.0–2.0) *1.6* (0.0–3.5) *6.2* (3.5–7.5) —	Go/No Go	All MS vs HS: lower deactivation RR vs CIS: greater activation, raising with cognitive demand SP vs CIS: idem	Positive correlation with EDSS	Positive correlation with BV; negative with T2LL
Colorado et al. (2012) [96]	23 RR 28 HS	*41.8* (9.9) *38.1* (12.5)	*7.4* (6.7) —	0.0 (0.0-1.5) —	Checkerboard, 4-finger flexion extension, N-back (*n* = 0.2)	Greater activation for N-back and for nondominant hand movement	—	Positive correlation with T2-LL in both right and left motor tasks
Hulst et al. (2012) [38]	34 CP 16 CI 30 HS	*46.0* (9.2) *50.3* (5.6) *44.5* (8.8)	*11.4* (6.6) *12.5* (7.3) —	*4.1* (1.3) *4.3* (1.5) —	Episodic memory encoding	CP: greater activation in the hippocampal memory system CI: lower activation in the hipp.	—	—
Kern et al. (2012) [39]	18 RR 16 HS	*42.1* (23.0–54.5) *35.2* (24.0–50.3)	3.0 (1.0–5.0) —	*1.7* (1.0–3.0)	Verbal task (encoding, recall)	Greater activation in the L anterior hipp. (cornu ammonis) and bilateral ento- and perirhinal cortices	Positive correlation with overall verbal memory performance	Positive correlation with fornix FA
Smith et al. (2012) [97]	12 MS 12 HS	*43.1* (8.5) *43.1* (9.8)	— —	<3 —	Information processing (semantic, choice)	Greater activation the DLPFC, PCC, R STG, and R TP; *lower* in the L MTG, L STG, R SMA, and R IPL Additional areas in choice condition	—	**—**
Forn et al. (2013) [98]	18 CIS 15 HS	*33.0* (8.8) *32.3* (7.2)	— —	1.5 (0.0–3.5)	SDMT	Greater deactivation of the R posterior cingulate gyrus	—	Positive correlation with T2-LL
Rocca et al. (2014) [36]	42 MS 52 HS	*39.6* (8.5)	*7.7* (2.0–15.0)	2.0 (1.0–4.0)	N-Back (*n* = 0, 1, 2, 3)		Negative correlation with disease duration; *positive* with cognitive performance	Negative correlation with T2-LL
Weygandt et al. (2017) [37]	18 high LL 12 low LL 21 HS	*49.8* (7.7) *45.0* (9.9) *49.1* (11.7)	*11.7* (7.2) *5.8* (4.0)	4.0 (2.5-6.0) 2.5 (1.5-6.0)	Decision making (Iowa gambling task, choice, and feedback conditions)	Greater activation in both NA-BT and affected areas for high LL None for low LL	**—**	**—**
Tacchino et al. (2018) [99]	17 CIS 20 RR 20 HS	*35.5* (8.16) *39.1* (9.5) *34.0* (8.1)	*14.1* (8.2) *2.3* (1.3) —	1.0 (0.0–2.0 1.5 (1.0–3.5) —	Mental (vs actual) movement	Greater activation in CIS vs RR or HS and in RR vs HS	Positive correlation with mental performance in the MS group and RR; *negative* in CIS	—

^∗^fMRI main results are reported with reference to the patient group, unless specified otherwise. MS: multiple sclerosis patients; PP: primary progressive MS; SP: secondary progressive MS; RR: relapsing-remitting MS; CIS: clinically isolated syndrome; BMS: benign MS; HS: healthy subjects; CI: cognitively impaired; CP: cognitively preserved; CC: corpus callosum; CG: cingulate gyrus; ACC: anterior cingulate cortex; IPL: inferior parietal lobule; hipp.: hippocampus; MFG: medial frontal gyrus; PCC: posterior cingulate cortex; PFC: prefrontal cortex; DLPFC: dorsolateral PFC; STG: superior temporal gyrus; SMC: sensorimotor cortex; SPG: superior parietal gyrus; SMA: supplementary motor area; SII: secondary sensorimotor cortex; TP: temporal pole; L: left; R: right; PASAT: Paced Auditory Serial Addition Task; PVSAT: Paced Visual Serial Addition Task; SDMT: Symbol Digit Modalities Test; EDSS: expanded disability status scale; DF: dominant foot; NDF: nondominant foot; DH dominant foot; NDH: nondominant hand; FA: fractional anisotropy; MD: mean diffusivity; MTR: magnetization transfer rate; FC: functional connectivity; EC: effective connectivity (assessed with dynamic causal modelling); NA: normal appearing; GM: grey matter; WM: white matter; BT: brain tissue; BV: brain volume; LL: lesion load; T1-LL: T1 lesion load; T2-LL: T2 lesion load; LI: lateralization index.

**Table 2:** Longitudinal t-fMRI papers.

Authors (year)	Sample size	Age at baseline	MS duration at baseline (years)	EDSS at baseline	Study design	Follow-up (months)	Functional main result(s)	Clinical correlation(s)	Structural correlation(s)
Pantano al. (2005) [46]	18 MS 9 HS	*31.0* (8.0) *31.0* (8.0)	— —	1.0 [0.0–2.5] —	Finger opposition task	15–26	Decreased activity in the ipsilateral SMC and contralateral cerebellum	Negative correlation with age and occurrence of new relapses	Correlation with lesion load changes at follow-up
Mezzapesa et al. (2008) [47]	5 RR	*35.1* [18.0–63.0] *38.6* [21.0–54.0]	*2.5* (3.2) —	1.5 [1.0–4.0] —	Four-finger flexion extension	6	Task execution with unimpaired hand reduced activation of the ipsilateral SMC, SMA, and contralateral SII	Reduced activation of the motor cortex only in patients with good recovery	—
Audoin et al. (2008) [48]	13 CIS 19 HS	*29.5* (6.0) *25.8* (6.0)	*0.5* (0.2) —	1.0 [0.0–2.0] —	PASAT	12	MS with decreased/unchanged PASAT: decrement of frontal activation MS with increment in PASAT score: increment of frontal activation	Positive correlation between change in PASAT performance and change in activation of the lateral prefrontal cortex	—
Pantano et al. (2011) [100]	19 RR relapse 13 RR stable	*36.8* (6.7) *37.4* (9.2)	*7.8* (6.7) *7.3* (5.6)	1.5 [0.0–3.5] 1.5 [1.0–3.0]	4-finger flexion extension	1-2	Greater deactivation in IPG activity in relapsing vs stable MS	Greater activity changes in fast vs slow recovery	—
Loitfelder al. (2014) [101]	13 RR 15 HS	*31.3* (10.0) *26.3* (4.7)	*2.55* [0.3–10.1] —	1.5 [0.0–3.5] —	Go/No Go task	20	Increased activation in L IPL	Negative correlation with SDMT and EDSS	Negative correlation with lesion load

SMC: sensorimotor cortex; SMA: supplementary motor area; SII: secondary sensory/sensorimotor cortex; IPG: inferior parietal gyrus; IPL: inferior parietal lobule. L: left; R: right; italic font: mean; round parenthesis: standard deviation; squared parenthesis: range.

**Table 3:** Interventional t-fMRI papers.

Authors (year)	Sample size	Age	MS duration (years)	EDSS	Study design	Intervention(s) (setting and schedule)	Functional main result(s)	Clinical correlation(s)	Structural correlation(s)
Parry et al. (2003) [49]	10 MS 11 HS	*41.0* [31.0–54.0] *42.0* [26.0–61.0]	*10.0* [5.0–21.0] —	2.0 [0.0–6.0] —	STROOP	Rivastigmine administration	Decrement of activation ratio	Correlation with STROOP and phonemic verbal fluency	Correlation with brain volume
Mainero et al. (2004) [50]	12 RR	—	—	—	Finger index to thumb opposition	Single oral dose of 3,4-diaminopyridine	Greater activation in the ipsilateral SMC and SII	Positive correlation between intracortical facilitation at TMS and activation of the ipsilateral SMC	—
Morgen et al. (2004) [52]	9 MS	*43.1* (10.4)	*9.6* (9.0)	2.0 [1.0–6.0]	Thumb flexion and thumb extension	30 minutes of thumb flexion training	MS patients did not show task-specific decreases in activation in the contralateral SMC, PMC, and IPL, as evidenced in HS	No significant results	No significant results
Rasova et al. (2005) [102]	17 MS active 11 MS control 13 HS	— — —	— — —	— — —	Finger index to thumb opposition	2-month neurophysiological, sensorimotor learning and adaptation, rehabilitative therapy	Increased correlation between activities in the L and R hemispheres	No significant results	—
Sastre-Garriga et al. (2010) [54]	15 MS 5 HS	*50.7* (10.9) —	*14.4* (8.9) —	6.0 [3.5–7.0] —	PASAT	5-week computer-based cognitive rehabilitation	Increased activity in several cerebellar areas	No significant results	—
Cerasa et al. (2013) [53]	12 RR active 11 RR control	*31.7* (9.2) *33.7* (10.3)	*4.3* (3.0) *5.1* (5.2)	3.0 [1.0–4.0] 2.0 [2.0–4.0]	PVSAT	Cognitive computer-assisted training	Greater activity in the R posterior cerebellar and L superior parietal lobules	Positive correlation with changes in STROOP score	—
Ernst et al. (2012) [55]	4 RR active 4 RR control	*37.3* (5.5) *39.8* (5.1)	*15.0* (9.3) *13.5* (7.2)	1.5 [0.0–4.0] 2.5 [0.0–4.0]	Autobiography and episodic memory	6 weeks Mental visual imagery cognitive training	Higher activation in posterior cortical areas	Association between increased activation of posterior regions and autobiographical memory	—
Tomassini et al. (2012) [2]	19 RR, 4 SP 12 HS	*45.0* (8.5) *43.0* (2.7)	*12.0* (1.5)	4.0 [0.0–7.0]	Visuomotor task	At least 15 days of the visuomotor task	Reduction in cortical activation in a greater number of cortical areas in MS than in HS	Negative correlation between performance improvement and occipital activation in HS but not in MS	—
Hubacher et al. (2015) [56]	6 RR active 4 RR control	*47.5* (6.4) *44.8* (5.6)	*2.5* (1.4) *4* (2.7)	2.0 [1.0–3.5] 1.0 [1.0–2.5]	N-Back (*n* = 1, 2, 3)	Computer-based cognitive training	Different and opposed changes in activation after rehabilitation	—	—
Tomassini al. (2016) [51]	26 MS 22 HS	*36.1* (1.4) *33.5* (1.7)	1.8 (0.5) —	1.5 [0.0–3.0] —	Two runs of thumb flexion separated by 25 minutes of training	12 weeks INF beta therapy	Reduction of between-run signal changes in secondary visual areas and motor, temporal, and parietal cortical areas	—	—
Leonard et al. (2017) [103]	7 MS active 7 MS control	*47.7* (28.0–61.0) *49.7* (38.0–62.0)	11.2 (3.0–26.0) 22.3 (9.0–37.0)	4.2 [3.0–6.0] 4.8 [3.0–6.0]	Gait Imagery Working memory	14-week cognitive rehabilitation combined with tongue stimulation	Gait Imagery: increment of L motor and premotor cortex activity Working memory: increment of activation of the left DLPFC	—	—

SMC: sensorimotor cortex; SII: secondary sensory/sensorimotor cortex; PMC: premotor cortex; IPL: inferior parietal lobule; DLPFC: dorsolateral prefrontal cortex. L: left; R: right; italic font: mean; round parenthesis: standard deviation; squared parenthesis: range.

**Table 4:** Cross-sectional r-fMRI papers.


Authors (year)	Sample size	Age (mean)	MS duration (years)	EDSS median	Technique(s)	Functional main result(s)	Clinical correlation(s)	Structural correlation(s)
Rocca et al. (2010) [83]	24PP 33 SP 24 HS	*47.9* [29.0–64.0] *46.3* [24.0–65.0] *47.4* [26.0–65.0]	*12.7* [3.0–39.0] *15.5* [4.0–32.0] —	6.0 [3.0–8.0] 6.0 [4.0–9.0]	ICA (DMN)	Reduction of RS activity in the ACC was more pronounced in cognitively impaired vs cognitively preserved patients	Positive correlation between PASAT and word list test scores and DMN abnormalities	Positive correlation with DTI changes in the corpus callosum and cingulum
Roosendaal et al. (2010) [60]	14 CIS 31 RR 41 HS	*34.6* (8.4) *39.1* (9.0) *38.6* (10.5)	1.4 [0.8–1.5] 3.5 [1.1–8.5]	2.0 [1.0–2.6] 2.5 [2.0–3.5]	ICA	Greater FC in many RSNs in CIS but not in MS with respect to HS	**—**	FC in several networks decreases with increasing structural damage
Bonavita et al. (2011) [63]	18 CP RR 18 CI RR 18 HS	*40.5* (6.9) *40.9* (8.7) *39.0* (10.0)	*10.9* (4.7) *11.9* (7.1) —	*2.6* (1.7) *2.8* (1.1) —	ICA DMN investigation	Decreased FC in the anterior component of the DMN	Negative correlation between global cognitive scores and FC in the tDMN	No significant result
Liu et al. (2011) [58]	35 RR 35 HS	*38.1* [18.0–58.0] *35.6* [18.0–54.0]	3.6 [0.5–17.0] —	2.5 [1.0–6.0]	ALFF	Increment ALFF in the bilateral thalami, R insula, and R superior temporal gyrus	Correlation between EDSS and ALFF in the R insula and R superior temporal gyrus	No significant results
Faivre et al. (2012) [61]	13 early RR 14 HS	*31.8* (7.4) *30.1* (8.6)	*1.1* [0.3–3.3] —	1.0 [0.0–3.0] —	ICA	Greater FC in the visual processing network, anterior DMN, dorsal FPN, prefronto-insular network, R ventral FPN, and R SMN	Negative correlation between 9-HPT and MSFC scores and FPN FC	No significant results
Liu et al. (2012) [57]	37 CIS 37 HS	*30.6* [15.0–56.0] *31.4* [17.0–55.0]	*0.3* [0.03–0.5] —	3.0 [1.0–6.0] —	ALFF	Decrement of ALFF in the R anterior cingulate cortex, caudate, lingual gyrus, and cuneus	No significant results	No significant results
Gallo et al. (2012) [65]	16 RR nON 14 RR ON 15 HS	*37.8* [19.0–51.0] *33.8* [21.0–50.0] *36.3* [20.0–53.0]	*10.0* [5.0–18.0] *8.2* [3.0–22.0] —	*2.0* [1.0–4.0] mean *2.1* [1.0–5.5] mean	ICA (visual network)	Lower FC in peristriate cortices, along the fusiform gyri, bilaterally. ON compared to nON: greater FC in the R lateral middle occipital gyrus and lower FC in the R lingual gyrus	Number of optic neuritis associated to reduction of FC in the R inferior peristriate cortex	No significant results
Schoonheim et al. (2012) [62]	12 RR 3 SP M 14 RR 1 SP F 15 HS M 15 HS F	*42.0* (9.6) *42.8* (9.6) *44.3* (8.2) *42.8* (7.6)	*5.1* (4.1) *4.9* (3.9) — —	*3.7* (1.9) *3.2* (1.1) — —	Graph theory ICA	More altered metrics in male MS	Positive correlation with performance	—
Schoonheim al. (2014) [71]	112 RR, 7 PP, 9 SP 50 HS	*40.4* (11.1) *41.0* (8.8)	*7.7* (2.2) —	2.0 [0.0–8.0] —	Fast eigenvector centrality mapping	Modulation of eigenvector centrality mapping and FC of SMC	Positive correlation with cognitive performance Negative correlation with EDSS	Correlation with thalamic volume
Tona et al. (2014) [67]	48 RR 24	*36.7* (8.1) *31.1* (6.5)	*7.4* (6.1) —	2.0 [1.0–4.5] —	Seed (thalamus)	Coexistence of areas of increased FC and areas of decreased FC	Negative correlation with PASAT 2 s/3 s	—
Zhou et al. (2014) [59]	13 RR 13 HS	42.1 [20.0–58.0] 41.8 [21.0–58.0]	*2.1* [0.1–12.5] —	1.5 [1.0–2.5] —	ALFF (thalamus)	Increment of ALFF in the bilateral thalami	Correlation with PASAT	Correlation with FA in the left thalamus
Liu et al. (2015) [68]	35 RR 35 HS	38.1 (18–58) 35.6 (18–54)	3.6 [0.5–17.0]	2.5 [1.0–6.0] —	Seed (thalamus)	Decreased FC between the thalamus and several brain regions	Negative correlation between disease duration and interthalamic FC	No significant results
Liu et al. (2016) [66]	28 RR 20 CIS 28 HS	*34.6* (10.0) *32.8* (12.3) *31.6* (11.4)	*3.3* (2.8) *0.3* (0.1) —	*2.7* (1.4) *3.1* (1.9)	nFCS	Increment of nFCS in CIS evolved in MS vs the remaining CIS in the R anterior cingulate and fusiform gyri	Positive correlation with EDSS	Positive correlation with lesion load
Rocca et al. (2016) [70]	246 MS 55 HS	*41.7* [20–60] *42.3* [19–60]	*13.7* [0.0–36.0]	3.0 [0.0–9.0]	Graph theory	Different characteristics and distribution of hubs	Association between cognitive deficits and impairment of global integration	No significant results.
Zhong et al. (2016) [69]	26 MI 17MP 20HS	*49.9* (12.25) *48.2* (8.6); *44.2* (13.3)	*12.8* (9.8) *7.4* (5.0) —	2.0 [0.0–6.0] 3.5 [0.0–6.5] —	Seed (LM1)	MI had weaker FC at LM1 with SMC and SII	Negative correlation between FC and 9HPT	Structural abnormalities in regions of FC impaired
Bisecco et al. (2017) [64]	28 RR F 31 RR NF 29 HS	*40.5* [21–62] *39.6* [23–54] *40.1* (10.1)	*13.8* [1.0–44.0] *11.2* [1.0–27.0] —	2.0 [1.0–5.5] 1.5 [1.0–6.0]	ICA (DMN, SMN)	Functional rearrangement of the DMN and SMN in the MSF	Association of fatigue severity and changes in DMN	—

FPN: frontoparietal network; SMN: somatomotor network; ON: MS with optical neuritis; nON: MS without optical neuritis; nFCS: normalized voxel-based functional connectivity strength. L: left; R: right; italic font: mean; round parenthesis: standard deviation; squared parenthesis: range.

**Table 5:** Longitudinal r-fMRI papers.

Authors (year)	Sample size	Age at baseline (mean)	MS duration (years)	EDSS (median)	Technique(s)	Follow-up	Functional main result(s)	Clinical correlation(s)	Structural correlation(s)
Droby et al. (2016) [72]	9 RR	*42.3* (9.2)	*6.2* (8.0) *1.0* (0.3)		Seed (left precuneus)	5 bimonthly follow-up periods	Higher FC in MS with periventricular lesion in the cuneus and precuneus regions No changes in FC over time in clinically stable RR	—	—
Faivre et al. (2016) [73]	38 RR 24 HS	*36.1* (5.1) *33.1* (6.4)	*10.0* (2.7) —	1.0 [0.0–6.5] —	Graph theory	2 years	Connectivity metric increment in not disabled and decrement in disabled MS	Correlation between decrease in connectivity metrics and disability progression	No significant results

Italic font: mean; round parenthesis: standard deviation; squared parenthesis: range.

**Table 6:** Interventional r-fMRI papers.

Authors (year)	Sample size	Age (mean)	MS duration (years)	EDSS (median)	Intervention(s) (setting and schedule)	Technique(s)	Functional main result(s)	Clinical correlation(s)	Structural correlation(s)
Filippi al. (2012) [75]	10 RR Active 10 RR Control	*46.7* [25.0–64.0] *44.8* [28–60]	*13.5* [1.0–28.0] *15.5* [1.0–28.0]	2.0 [1.5–4.0] 2.5 [1–4.0.0]	12 weeks of computer-assisted cognitive rehabilitation	ICA	FC in the DMN, SPN, and EFN increase or stay stable in the active group	FC changes correlates with cognitive improvement	—
Petsas et al. (2015) [74]	20 RR 14 HS	*34.0* (6.0) *31.0* (5.0)	*13.5* (16.5) —	1.5 [0.0–3.0] —	25 minutes of right repetitive thumb flexions	ICA	Greater increment of FC in the cerebellum	No significant results	Correlation between FC in SMN and lesion volume
Boutiére et al. (2016) [77]	9MS Active 7 SP, 8 MS Control	*48.2* (9.4) *55.4* (11.1)	*12.2* (8.2) *18.7* (11.0)	6.0 [4.0–7.0] 6.0 [6.0–6.5]	13 consecutive days of theta burst stimulation of the motor cortex.	Graph theory	Laterality increases at the end of stimulation and returns to baseline after two weeks	Positive correlation with improvement of spasticity	—
De Giglio et al. (2016) [76]	11 MS Active 11 MS control	*42.0* (8.8) *41.1* (4.4)	*12.9* (5.7) —	2.0 [2.0–7.0] —	8-weeks of video game-based cognitive rehabilitation	Seed (thalamus)	Increase FC in the posterior cingulate, precuneus, and parietal cortex and decrement of FC in the cerebellum and L DLPFC	Positive correlation of FC in the parietal cortex and cognitive improvement	—

SPN: salience processing network; EFN: executive function network; DLPFC: dorsolateral prefrontal cortex. L: left; italic font: mean; round parenthesis: standard deviation; squared parenthesis: range.

**Table 7:** Quality assessment.

	Q1	Q2	Q3	Q4	Q5	Q6	Q7	Q8	Q9	Q10	TOT
*t-fMRI cross-sectional*											
*Sensorimotor*											
Reddy et al. (2000) [22]	1	0	1	1	0	0	0	0	1	0	4
Filippi et al. (2002) [16]	0	1	1	0	0	1	0	0	1	0	4
Pantano et al. (2002) [19]	1	0	1	1	0	1	0	1	1	0	6
Reddy et al. (2002) [13]	0	0	1	1	0	1	1	1	1	1	7
Pantano et al. (2002) [8]	0	1	1	1	0	1	0	1	1	0	6
Rocca et al. (2002) [17]	1	1	1	0	0	1	0	0	1	0	5
Rocca et al. (2003) [14]	0	0	1	0	0	1	0	0	1	0	3
Rocca et al. (2003) [9]	1	1	1	1	0	1	0	0	1	0	6
Rocca et al. (2003) [80]	1	0	1	1	0	1	0	0	1	0	5
Filippi et al. (2004) [12]	0	1	1	1	0	1	0	0	0	0	4
Filippi et al. (2004) [86]	1	1	1	1	0	1	0	0	1	0	6
Rocca et al. (2005) [20]	1	1	1	1	0	1	0	1	0	0	6
Ciccarelli et al. (2006) [18]	0	0	1	1	0	1	1	1	1	1	7
Wang and Hier (2007) [87]	0	0	1	0	1	0	0	0	1	1	4
Wegner et al. (2008) [88]	0	1	0	1	0	1	1	1	0	1	6
Rocca et al. (2009) [79]	0	1	0	1	0	1	1	1	1	1	7
Harirchian et al. (2010) [10]	0	1	1	1	0	1	0	0	0	0	4
Rocca et al. (2010) [15]	1	1	1	1	1	1	0	1	1	0	8
Rico et al. (2011) [11]	0	0	1	0	0	1	0	0	1	0	3
Petsas et al. (2013) [21]	1	1	1	1	0	1	1	0	1	1	8
Faivre et al. (2015) [89]	0	0	1	1	1	1	1	1	0	1	7
*Cognitive*											
Staffen et al. (2002) [23]	0	1	1	0	0	0	0	0	0	0	2
Audoin et al. (2003) [24]	1	0	1	1	0	0	0	1	1	0	5
Penner et al. (2003) [45]	1	0	1	1	0	1	0	0	0	0	4
Mainero et al. (2004) [26]	0	1	1	0	0	1	1	1	1	0	6
Saini et al. (2004) [90]	0	0	1	1	0	1	0	1	1	1	6
Audoin et al. (2005) [25]	0	1	1	0	0	1	0	0	1	0	4
Cader et al. (2006) [31]	1	1	1	0	1	1	1	1	1	0	8
Forn et al. (2006) [27]	0	0	1	0	0	0	0	0	0	0	1
Rachbauer et al. (2006) [28]	0	1	1	0	0	0	0	0	0	0	2
Sweet et al. (2006) [32]	1	1	1	0	0	1	0	0	1	1	6
Forn et al. (2007) [33]	1	0	1	0	0	0	0	0	0	1	3
Morgen et al. (2007) [42]	1	1	1	1	0	1	1	1	1	0	8
Nebel et al. (2007) [91]	0	0	1	1	0	1	0	0	0	1	4
Prakash et al. (2007) [29]	1	0	1	0	1	1	0	1	0	1	6
Prakash et al. (2008) [43]	1	1	1	0	1	1	0	1	0	1	7
Bonzano et al. (2009) [30]	0	0	1	1	0	1	0	0	0	0	3
Passamonti et al. (2009) [44]	1	0	1	1	0	1	1	0	0	0	5
Pierno et al. (2009) [92]	1	1	1	1	0	1	1	0	0	0	6
Rocca et al. (2009) [81]	1	1	0	1	1	1	0	1	1	1	8
Smith et al. (2010) [93]	1	0	1	0	0	1	1	0	0	1	5
Bonnet et al. (2010) [40]	0	1	1	1	0	0	1	1	1	0	6
Helekar et al. (2010) [94]	1	1	1	0	1	1	0	1	0	0	6
Rocca et al. (2010) [34]	1	1	1	0	1	1	0	1	1	0	7
Amann et al. (2011) [35]	0	1	1	1	1	1	0	0	0	0	5
Jehna et al. (2011) [95]	1	1	1	1	1	1	0	0	1	0	7
Loitfelder et al. (2011) [41]	0	1	1	0	1	1	1	1	1	1	8
Colorado et al. (2012) [96]	1	1	1	1	1	1	0	0	1	1	8
Hulst et al. (2012) [38]	0	1	1	0	0	1	1	0	0	0	4
Kern et al. (2012) [39]	0	1	1	1	1	1	0	1	1	1	8
Smith et al. (2012) [97]	1	0	1	0	0	1	0	0	0	0	3
Forn et al. (2013) [96]	1	1	1	1	1	1	0	0	1	0	7
Rocca et al. (2014) [36]	0	1	1	1	1	1	1	1	1	0	8
Weygandt et al. (2017) [37]	1	1	0	1	1	1	0	1	0	0	6
Tacchino et al. (2018) [99]	0	1	1	1	0	1	1	1	0	1	7
*t-fMRI longitudinal*											
Pantano et al. (2005) [46]	0	0	0	1	0	1	0	1	1	1	5
Mezzapesa et al. (2008) [47]	0	0	1	1	0	1	0	1	0	1	5
Audoin et al. (2008) [48]	1	1	1	1	0	1	1	1	0	0	7
Pantano et al. (2011) [100]	1	1	1	1	0	1	1	1	0	1	8
Loitfelder et al. (2014) [101]	0	0	1	1	1	1	0	1	1	1	7
*t-fMRI Interventional*											
Parry et al. (2003) [49]	0	0	1	1	1	1	0	1	1	1	7
Mainero et al. (2004) [50]	0	0	1	1	0	1	0	1	0	0	4
Morgen et al. (2004) [52]	0	0	1	1	0	1	0	1	1	0	5
Rasova et al. (2005) [102]	1	1	0	1	0	1	0	1	0	0	5
Sastre-Garriga et al. (2011) [54]	0	0	0	0	0	1	0	1	0	1	3
Cerasa et al. (2013) [53]	0	0	1	1	1	1	1	1	0	1	7
Ernst et al. (2012) [55]	1	0	1	0	1	0	0	1	0	1	5
Tomassini et al. (2012) [2]	0	1	1	1	0	1	1	1	0	1	7
Hubacher et al. (2015) [56]	0	0	1	1	1	0	0	0	0	1	4
Tomassini al. (2016) [51]	1	1	0	1	1	1	1	0	0	1	7
Leonard et al. (2017) [103]	1	0	0	0	1	1	0	0	0	1	4
*r-fMRI cross-sectional*											
Roosendaal et al. (2010) [60]	1	1	1	0	0	1	1	0	1	0	6
Bonavita et al. (2011) [63]	0	1	1	1	1	1	0	1	1	0	7
Liu et al. (2011) [58]	0	1	1	0	0	1	1	1	1	0	6
Faivre et al. (2012) [61]	0	0	1	1	1	1	1	1	1	0	7
Liu et al. (2012) [57]	1	1	1	0	0	1	0	1	1	1	7
Gallo et al. (2012) [65]	0	1	1	1	1	1	0	1	1	1	8
Rocca et al. (2010) [83]	1	1	1	1	1	1	1	1	1	1	10
Schoonheim et al. (2012) [62]	0	1	1	0	0	1	1	1	0	1	6
Schoonheim al. (2014) [71]	0	1	1	1	1	1	1	1	1	1	9
Tona et al. (2014) [67]	0	1	1	1	1	1	1	1	0	1	8
Zhou et al. (2014) [59]	1	0	1	1	1	1	0	1	1	1	8
Liu et al. (2015) [68]	0	1	1	0	0	1	1	1	1	1	7
Liu et al. (2016) [66]	0	1	1	0	0	1	1	1	1	0	6
Rocca et al. (2016) [70]	1	1	1	1	1	1	1	1	1	1	10
Zhong et al. (2016) [69]	1	1	1	1	1	1	1	1	1	1	10
Bisecco et al. (2017) [64]	1	1	1	1	1	1	1	1	0	1	9
*r-fMRI longitudinal*											
Droby et al. (2015) [72]	0	0	1	0	1	1	1	0	0	1	5
Faivre et al. (2016) [73]	1	1	1	1	0	0	1	1	1	1	8
*r-fMRI interventional*											
Filippi et al. (2012) [75]	0	0	1	1	1	1	0	1	0	1	6
Petsas et al. (2014) [74]	1	1	1	1	1	1	1	1	1	1	10
Boutiere et al. (2016) [77]	1	0	1	1	1	0	0	1	0	1	6
De Giglio et al. (2016) [76]	0	1	1	1	1	1	1	1	0	1	8
Methodology	Q1: Were a priori hypotheses clearly stated?										
	Q2: Was the sample size equal to or larger than 30 subjects?										
Clinical characteristics	Q3: Was MS phenotype included?										
	Q4: Was clinical information, e.g., relapses and/or treatment, reported?										
MRI parameters	Q5: Was the used MRI filed equal to or greater than 3T?										
Statistical analysis	Q6: Was correction for multiple comparison used?										
	Q7: Were covariates of no interest included in the analysis?										
Results	Q8: Were correlations between fMRI outcomes and clinical scores investigated?										
	Q9: Were correlations between fMRI outcomes and structural measures investigated?										
	Q10: Were limitations of the study clearly stated?										
Yes = 1											
No = 0											
