# Erratum to “The Role of fMRI in the Assessment of Neuroplasticity in MS: A Systematic Review”

**DOI:** 10.1155/2019/5181649

**Published:** 2019-06-03

**Authors:** Laura De Giglio, Silvia Tommasin, Nikolaos Petsas, Patrizia Pantano

**Affiliations:** ^1^Department of Human Neurosciences, Sapienza University of Rome, Rome, Italy; ^2^Multiple Sclerosis Centre, Sant'Andrea Hospital, Sapienza University of Rome, Rome, Italy; ^3^IRCCS NEUROMED, Pozzilli, Italy

In the article titled “The Role of fMRI in the Assessment of Neuroplasticity in MS: A Systematic Review” [1], the authors' names were reversed. The corrected author's list is shown above.
